# *Escherichia coli* cells are primed for survival before lethal antibiotic stress

**DOI:** 10.1128/spectrum.01219-23

**Published:** 2023-09-12

**Authors:** Tahmina Hossain, Abhyudai Singh, Nicholas C. Butzin

**Affiliations:** 1 Department of Biology and Microbiology, South Dakota State University, Brookings, South Dakota, USA; 2 Electrical & Computer Engineering, University of Delaware, Newark, Delaware, USA; 3 Department of Chemistry and Biochemistry, South Dakota State University, Brookings, South Dakota, USA; University of Exeter, Exeter, United Kingdom

**Keywords:** antibiotic tolerance, antibiotic persistence, antibiotic resistance, Luria-Delbrück fluctuation test, epigenetic, heterogeneity

## Abstract

Non-genetic factors can cause significant fluctuations in gene expression levels. Regardless of growing in a stable environment, this fluctuation leads to cell-to-cell variability in an isogenic population. This phenotypic heterogeneity allows a tiny subset of bacterial cells in a population called persister cells to tolerate long-term lethal antibiotic effects by entering into a non-dividing, metabolically repressed state. We occasionally noticed a high variation in persister levels, and to explore this, we tested clonal populations starting from a single cell using a modified Luria-Delbrück fluctuation test. Although we kept the conditions same, the diversity in persistence level among clones was relatively consistent: varying from ~60- to 100- and ~40- to 70-fold for ampicillin and apramycin, respectively. Then, we divided and diluted each clone to observe whether the same clone had comparable persister levels for more than one generation. Replicates had similar persister levels even when clones were divided, diluted by 1:20, and allowed to grow for approximately five generations. This result explicitly shows a cellular memory passed on for generations and eventually lost when cells are diluted to 1:100 and regrown (>seven generations). Our result demonstrates (1) the existence of a small population prepared for stress (“primed cells”) resulting in higher persister numbers; (2) the primed memory state is reproducible and transient, passed down for generations but eventually lost; and (3) a heterogeneous persister population is a result of a transiently primed reversible cell state and not due to a pre-existing genetic mutation.

**IMPORTANCE:**

Antibiotics have been highly effective in treating lethal infectious diseases for almost a century. However, the increasing threat of antibiotic resistance is again causing these diseases to become life-threatening. The longer a bacteria can survive antibiotics, the more likely it is to develop resistance. Complicating matters is that non-genetic factors can allow bacterial cells with identical DNA to gain transient resistance (also known as persistence). Here, we show that a small fraction of the bacterial population called primed cells can pass down non-genetic information (“memory”) to their offspring, enabling them to survive lethal antibiotics for a long time. However, this memory is eventually lost. These results demonstrate how bacteria can leverage differences among genetically identical cells formed through non-genetic factors to form primed cells with a selective advantage to survive antibiotics.

## INTRODUCTION

Clonal populations often exhibit phenotypic heterogeneity, leading to specific physiological effects that distinguish some cells from others (1 – 3). Variability can slightly reduce fitness in a common environment but, in return, maximize it during environmental perturbations (2, 4). For example, bacterial persister cells, a phenotypic variant, can endure prolonged lethal antibiotic treatment by entering a metabolically repressed state (5). This small subpopulation can reestablish infection after treatment and necessitate repeated long-term antibiotic therapy. When persisters emerge, they also have a high mutation rate that increases the likelihood of evolving antibiotic resistance (6), a pressing public health concern. A recent study provides evidence that among infectious diseases, antibiotic resistance may be the leading cause of death worldwide (more than HIV or malaria) (7). Global death projections are now estimated at 10 million per year by 2050 (8, 9) unless new technologies are developed to combat them, and we have a better understanding of persister survival. Here, we specifically focus on how heterogeneity in a population drives persister numbers.

Noise or fluctuation in gene expression levels drives phenotypic variation that could be an inherent survival strategy of a clonal bacterial population (2, 10). Studies reported that stress-response genes are generally more variable than housekeeping genes (11, 12). However, it is uncertain whether this variability is controlled or an effect of inevitable stochastic fluctuations in gene expression (13). We formalize this notion and hypothesize that specific cells are prepared for stress through a transiently inherited cell state.

**Fig 1:**
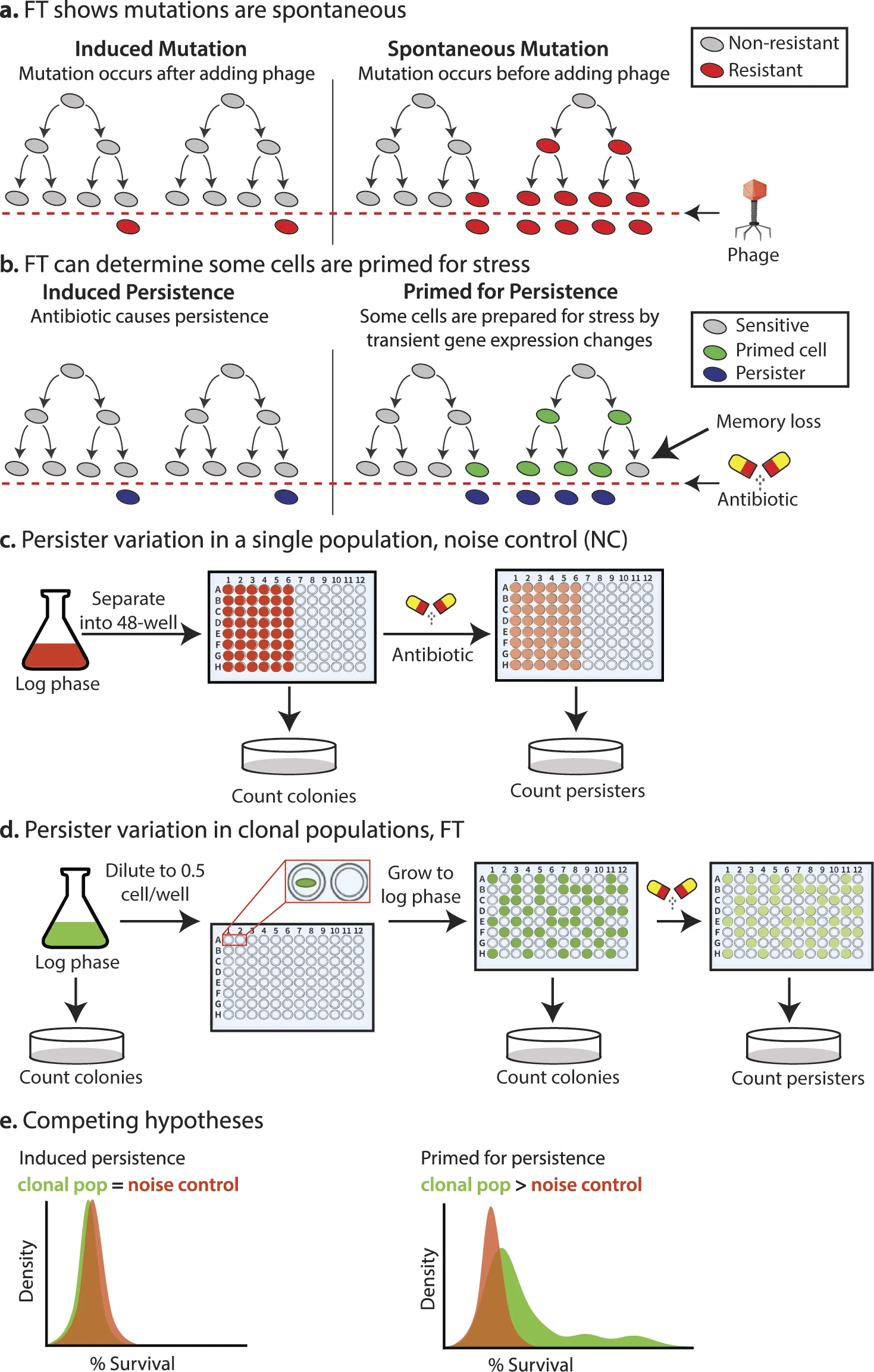
(a) The Luria-Delbrück FT. Each clone starts from a single cell and is then infected by a phage (virus). If resistance mutations are virus induced, the number of resistant cells would follow a Poisson distribution across clones. In contrast, if mutant cells arise spontaneously prior to viral exposure, there will be considerable clone-to-clone fluctuations in the number of surviving cells, including mutations that happen early in the lineage expansion causing many cells to be resistant. **(b**) FT to determine if some cells are primed for stress. This design follows the same experimental setup as (a), but it uses antibiotic stress and measures variation in persister cells (cells in a repressed cellular activity state by having transient gene expression change) across the clones. If persisters are antibiotic induced, then the number of persister cells would follow a Poisson distribution across clones. In contrast, if there are primed cells with a unique transient gene expression profile (caused by non-genetic factors) that allows them to prepare for stress before antibiotic exposure, there will be considerable clone-to-clone fluctuations in the number of surviving cells. Transient gene expression changes can be heritable and happen early in the lineage expansion, causing many cells to become primed for stress with a transient memory. **(c**) Persister variation check in a single population, referred to as noise control (NC). Cultures were grown to mid-log phase, ~1E + 8 CFU/mL, separated into 48 wells, treated with an antibiotic, 0.1 mg/mL Amp or Apr, and plated before and after antibiotic treatment to get percent survival. (d) Persister variation check in clonal populations started from a single cell, referred to as FT. Cultures were diluted to 0.5 cell/well and treated similarly to (a). **(e)** A model for competing hypotheses; *Induced persistence:* persisters are induced due to stress, and no difference will be observed in persister variation between clonal populations and noise control. *Primed persistence:* some cells are primed prior to stress; thus, some clonal populations will have more prime cells than others, and the persister variation will be higher in the clonal populations than in the NC.

In this work, we take advantage of the Luria-Delbrück fluctuation test (FT), which was recently used to identify genes related to cancer persistence against cancer drugs (14). Cancer persisters are similar in phenotype to bacterial persistence but biochemically unrelated. We used a similar approach to probe bacterial persistence. The FT was first pioneered ~80 years ago when Luria and Delbrück demonstrated that genetic mutations arise randomly in the absence of selection but not in response to the selection (15). They were studying phage (bacterial virus) infections. During that time, it was debated whether mutations leading to resistance were directly induced by the virus (Lamarckian theory) or if they developed randomly in the population before viral infection (Darwinian theory). They designed an elegant experiment where single cells were isolated and grown into clones and then infected by a phage (Fig. 1a). The number of resistant bacteria was counted across clones. Suppose mutations are virus induced (i.e., no heritable genetic component to resistance), where each cell has a small and independent probability of acquiring resistance. In this case, clone-to-clone fluctuations in the number of resistant cells should follow Poisson statistics (a memoryless process). In contrast, if mutations occur randomly before viral exposure (spontaneous mutation), the quantity of surviving bacteria will vary considerably across clones depending on when the mutation arose in the clone expansion. The data clearly showed a non-Poissonian skewed distribution for the number of resistant bacteria, validating the Darwinian theory of evolution (15) (Fig. 1a). This work led to a Nobel Prize, and the FT remains the most commonly used method to measure mutation rates in microbes (16).

While Luria-Delbrück focused on irreversible genetic alterations driving phage resistance, here, we use the FT to elucidate transient cell states that originate via reversible non-genetic mechanisms (14, 17 – 19). Notably, this generalized FT can be applied when single cells reversibly switch between drug-sensitive and drug-tolerant states even before treatment. Clone-to-clone fluctuations can be exploited to quantify these switching rates rigorously (20). This approach has been key in deciphering drug-tolerant cancer cells that arise stochastically even before drug exposure. Thus, cancer cells have a bet-hedging mechanism to survive sudden hostile extracellular environmental changes (14, 17, 18). Here, we are exploring “primed cells” (cells prepared for stress and arise by a rare, transiently inherited cell state) present before the treatment and prolonging survival once treatment begins (Fig. 1b through e).

In this study, we applied the FT to indicate some cells are prepared for stress and have an inherent transient memory. We applied this approach in conjunction with mathematical modeling to elucidate stochastic phenotype switching in response to antibiotic stress, an inherent survival strategy that gives flexibility to a clonal population. Our work demonstrates how heritable transient cell state changes can lead to variation in persister numbers. Exploring this phenomenon can shed light on how bacteria endure stress, a key question in persister research (21).

## RESULTS

### Primed cells are prepared for stress before antibiotic treatment

Bacterial populations are heterogeneous, even in log growth phase in a well-controlled lab environment and especially in natural systems (22, 23). The quantification of persister numbers often have huge variations with large error bars (SD or SEM), sometimes with hundreds of fold differences (24 – 26). We used defined media [MMB+ (27, 28)], which contains only chemically known components, to minimize variations between experiments. We optimized and standardized our experimental design and reduced the internal error rate to below twofold. Much of this error comes from the compounding imprecisions from multiple pipetting (all pipets have an error rate, and these experiments require serial dilutions). Despite reducing the error rate, we noticed that there would occasionally be huge outliers (up to 100-fold higher or lower than the average).

**Fig 2:**
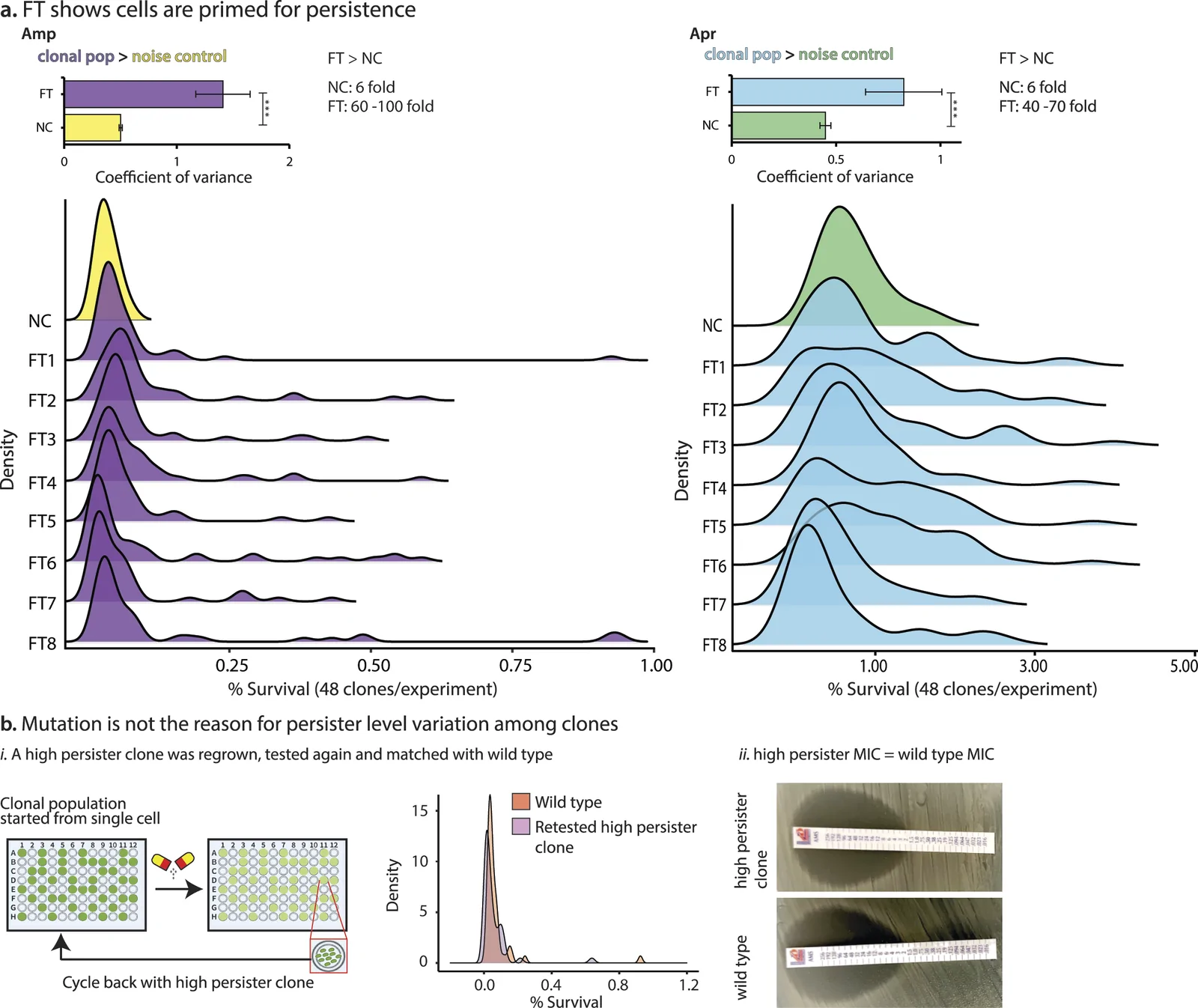
Some cells are prepared and primed for stress. (a) Results support primed persistence hypothesis: Population-level variation for Amp and Apr: approximately 6-fold (experimental setup described in Fig. 1c). FT from single cell level (experimental setup described in Fig. 1d): treatment with Amp or Apr shows 60- to 100-fold and 40- to 70-fold variation, respectively. **(b**) Mutations do not cause persister level variation among the clones. (**i)** FT with wild-type clonal populations and FT with clonal populations started from a high persister clone have a similar average persister level and persister range among 48 clones. (ii) Minimum inhibitory concentration (MIC) test showing no change in MIC level in high persister clones. ***P < 0.001.

We determined the typical persister variation in an *E. coli* population (noise control [NC]) is higher than our internal error. We grew a culture to mid-log phase and then divided it into 48 wells. We then treated it with ampicillin (Amp) or apramycin (Apr) for 3 h, followed by a persister assay (Fig. 1c). This resulted in a variation of approximately 6-fold for either antibiotic. These results do not explain the 100-fold changes that we occasionally observed. Unable to explain the variation at the population level, we wondered if a “memory” was passed down over several generations and skewed our data. We set out to test for variations from a single cell using an FT (14, 17, 18). Cultures were diluted to about 0.5 cell/well [Limiting Dilution assay (29, 30) in a 96-well microplate; on average, 48 wells had growth, and 48 wells did not (Fig. 1d)]. Cells were then grown to an optical density (OD) of 0.4–0.6 (log phase) and treated with Amp for 3 h. Most (but not all wells) reach the desired OD range simultaneously. We use two 96-well plates and pick 48 clones within the OD range to deal with this. After treatment, the cells were plated on Petri dishes and grown, and colonies were counted to get the colony forming unit per millimeter. We first tested lethal Amp dosages for 3 h, and the persister range was vast: ~60- to 100-fold. We tested this several times (Fig. 2a; eight separate experiments with ~48 clones/experiment), and there is consistently an extensive range of persister variation among the clones. We wondered if this was specific to Amp, so we tested another class of antibiotic, Apr. Amp targets the cell wall (31), while Apr targets the 30S ribosomal subunit (32). Again, the persister range was vast: ~40- to 70-fold range with Apr (Fig. 2a). It is not surprising that the range of survival for the two antibiotics is different, as Amp and Apr target distinct cellular processes, effect the cell differently, and kill at different rates. It is important to note that these results are not due to a carryover from stationary phase. The cultures were in exponential phase and diluted to a single cell, which was grown to exponential phase (~1E + 8 CFU/mL; ~OD 0.5). At this point, any remnants from stationary phase were lost.

This left two competing hypotheses to explain the extensive range in persistence: either (1) mutation* or (2) some cells are prepared for stress (“primed cells”), and these primed cells exhibit specific characteristics that allow them to prepare prior to the stress. *Although persisters are not caused by mutations, we needed to assess if a mutation causes this extensive persister range. We tested for antibiotic resistance by streaking clones on antibiotic plates, and no resistant colonies grew (Fig. S1a). To further test for mutations, we diluted the high persister clone (Hp clone) into 0.5 cell/well and repeated the FT. If an Hp clone was mutated, the average persister percentage would increase, and the range would decrease. However, they had the same average persister percentage and similar range (~60- to 100-fold) in both fluctuation experiments (Fig. 2b *i*). It is well established that a higher minimum inhibitory concentration (MIC, the minimal antibiotic required to hinder growth) corresponds with a higher resistance level, and persisters can survive in the presence of an antibiotic without an increase in the MIC after treatment (33). Indeed, models of resistance often define it as an increase in the MIC (33).

In addition, according to the guideline of studying persisters (33), the MIC level of persisters should be checked after the first round of antibiotic treatment. If the primed cells developed resistance, their MIC would change. However, the MIC remained the same as shown using a common clinical and laboratory (34) MIC test strip (Fig. 2b *ii*). These results undoubtedly ruled out mutations as the cause and led us to test Hypothesis 2, that is, some cells are primed prior to stress.

### Slight changes in cell density are not the primary factor for variation in persister number in exponential phase

Bacterial persistence levels could be controlled by density-dependent response. In addition, several studies showed that persister fraction increases sharply with increase of cell densities or when the population moved from mid-exponential to stationary phase (35, 36). We specifically tested persister levels in our FTs in exponential phase and not stationary phase to minimize the stationary phase effect. Before testing Hypothesis 2, we wanted to know how much cell density in exponential phase could skew our results by testing the overall persister range with slightly different cell densities using FTs. For FTs in Fig. 2a, cells were harvested at OD 0.4–0.6, since the persister levels are remarkably similar at these ODs. We detected no correlation between cell density and percent survival in 15 FTs (7 Amp and 8 Apr) and a weak correlation in FT3 with Amp treatment (Fig. S1b *i*). Thus, to further determine how much cell density affects the persister levels, we tested persister levels from OD 0.3–0.7 in ~0.1 OD intervals in a general population treated with 3 h Amp or Apr. At these ODs, cells are in exponential phase, and cell density ranges from ~2E07 to 2E08 CFU/mL. No appreciable correlation was observed with either antibiotic (Fig. S1b *ii*).

### Cells are primed for persistence, not short-term tolerance

**Fig 3:**
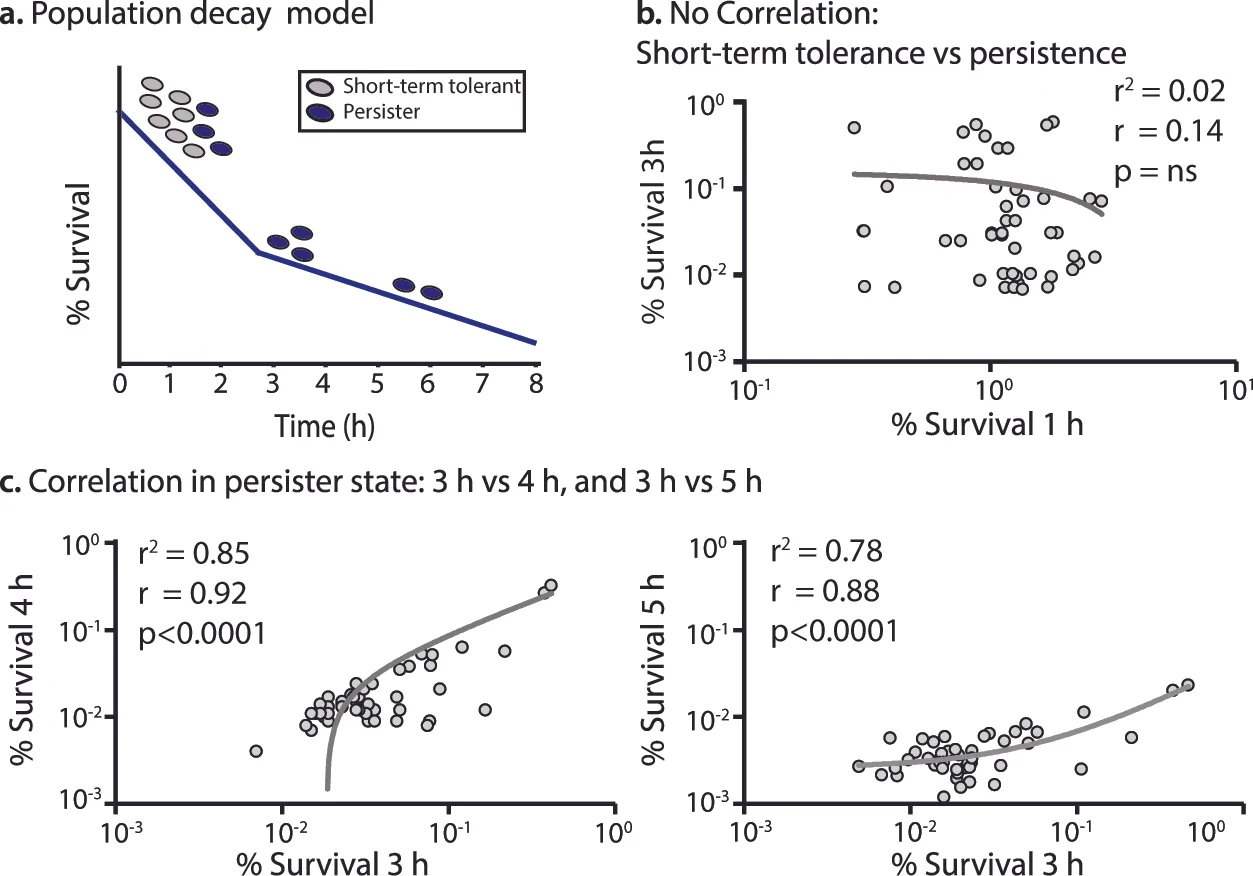
Cells are primed for persistence, not for short-term tolerance. (a) A simplified model of population decay indicates a biphasic death curve where susceptible cells or short-term tolerant cells die quicker than persisters. (b) Compare each clone’s short-term tolerance levels (1 h Amp) to their persister level (3 h Amp). (c) Compare each clone’s persister level at 3 h Amp to 4 h Amp (left) or 5 h Amp (right) (long-term persister level). About 48 clones were grown from a single cell and treated with Amp (0.1 mg/mL), and the percent survival was determined at different time points. Linear regression lines are shown in b-c. r, Pearson’s correlation coefficient; ns, not significant.

Short-term tolerance can mask persistence, and experimental evidence has shown that the phenotypes are distinct from each other (37). Short-term tolerant cells are dividing and likely have different survival mechanisms than persisters. In the initial stage of antibiotic treatment, there are far more short-term tolerant cells than persister cells (Fig. 3i). We did a similar FT with clones grown from a single cell and treated them with a lethal Amp concentration (0.1 mg/mL). Percent survival was determined for 1 h and 3 h of treatment using persister assays (27, 28, 38). Short-term tolerance at 1 h does not indicate the level of persistence at 3 h with lethal Amp; no correlation at 1 h vs 3 h population (*r^2^* = 0.02). If the primed cells are advantageous for long-term survival, we expect the high persister populations observed at 3 h treatment to stay high with more prolonged antibiotic exposure. As expected, percent survival at 3 h highly correlates with percent survival at 4 h (*r^2^* = 0.85) and 5 h (*r^2^* = 0.78) in Amp-treated populations (Fig. 3). Therefore, our results demonstrate that cells are primed for persistence and not for short-term tolerance.

### Primed cells have transient memory

**Fig 4:**
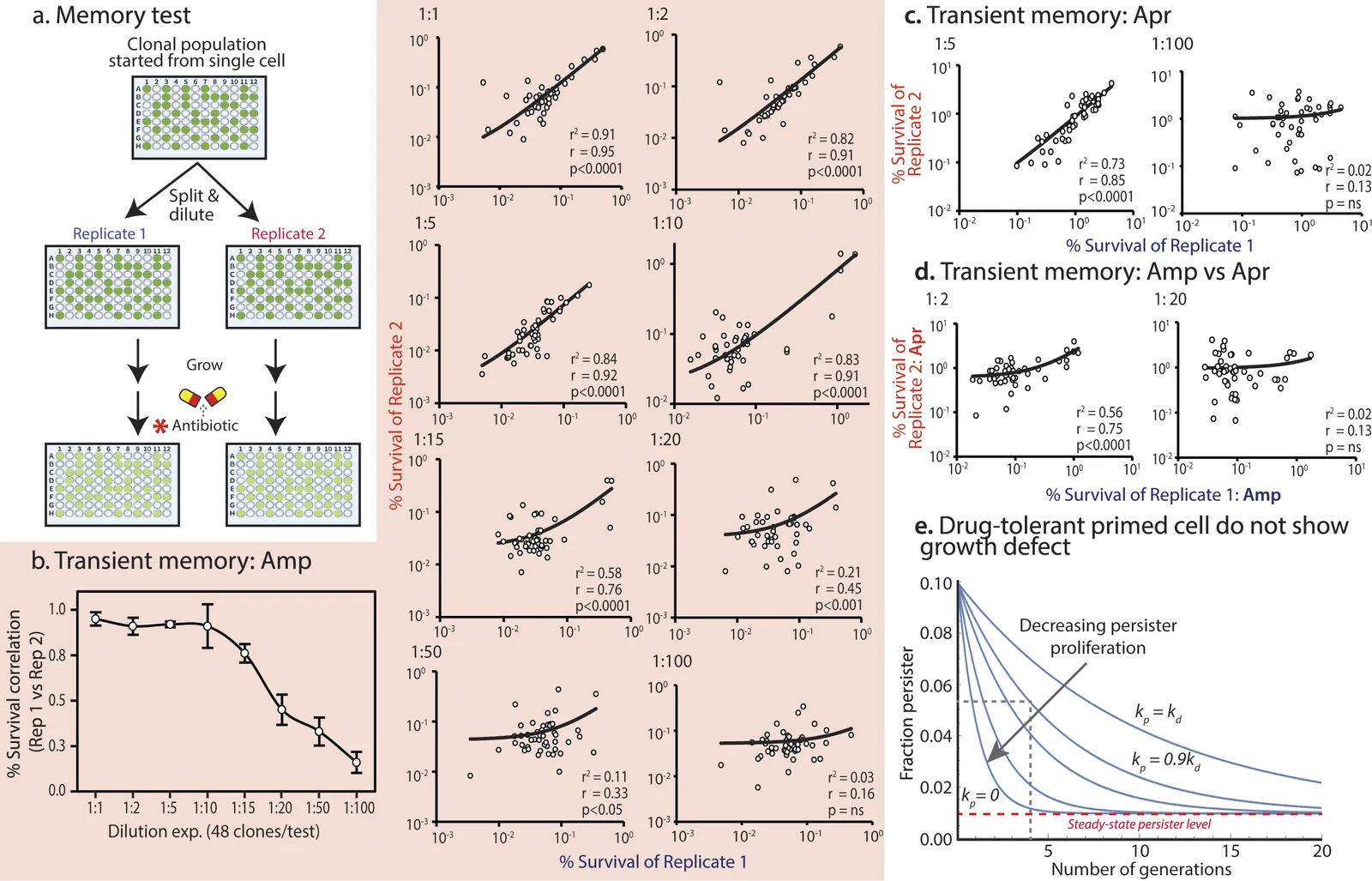
Prime cells have transient memory. (a) FT setup to check prime cell’s memory: cultures were diluted to 0.5 cell/well, grown to mid-log phase (~1E + 8 CFU/mL), each clone is divided and diluted into two replicates, grown to mid-log, and then tested for persistence. (b and c) Prime cells have transient memory for several generations before 3-h Amp and 3-h Apr treatment. The persister levels correlate even with 1:20 dilutions between Replicates 1 and 2, suggesting a strong memory within the primed subpopulation. The memory is eventually lost with a 1:100 dilution. (d) Prime cells show a weak memory when split and tested with Amp vs Apr. *Antibiotic: Amp vs Amp (replicates 1 and 2 both treated with Amp) or Apr vs Apr (replicates 1 and 2 both treated with Apr) or Amp vs Apr (replicates 1 and 2 treated with Amp and Apr, respectively). Axes are percent survival. r, Pearson’s correlation coefficient; ns, not significant. (**e**) Plot representing the fraction of persisters $\frac{y(t)}{x(t)}$ as predicted by the differential equation model (see Methods), assuming that initially 10% of cells where persisters and the steady-state persister level was $f_{s}=1\%$ . The five lines are plotted assuming the persister proliferation rate, $k_{p}=k_{d},0.9k_{d},0.8k_{d},0.5k_{d}$ , 0 where $k_{d}=1$ and $k_{2}=k_{p}/10$ . The black dashed lines show the numbers of generations it takes for $\left(t\right)=\frac{f\left(0\right)+f_{s}}{2}$ , when $k_{p}=0.9k_{d}$ .

Next, we determined whether high persister clones arise randomly due to noise in gene expression levels or in rare events where the gene expression level (memory) is passed down for several generations. We hypothesize that there is a transient memory at the transcriptomic level. The null hypothesis is that there is no memory and the variation range in persistence is random and solely due to noise. To test this, we divided and diluted the culture between 1:1 and 1:100 into separate microplates and allowed them to grow (Fig. 4a). If the null hypothesis is correct, there should be no correlation between divided cultures. However, persister levels were strongly correlated until the 1:20 dilution, and the memory is completely lost after a 1:100 dilution, supporting our transient memory hypothesis (Fig. 4b). We hypothesize that the memory and loss of memory that we observe with Amp could also be observed with another class of antibiotics. If so, this would show that transient memory is important to cell survival for different types of stress. We further confirmed the transient memory hypothesis with Apr as we see memory when cells are diluted 1:5, but memory is lost when diluted 1:100 (Fig. 4c). These results also add additional support that primed cells are not mutants because 1:100 dilution led to no long-term (genetic) survival phenotype (in several different clones), as a resistance mutation would allow.

We hypothesize that the same primed cells will allow higher persister levels when using antibiotics from classes that target distinct cellular processes, e.g., Amp and Apr. If both Amp and Apr primed cells use an akin mechanism, we expect a reasonable correlation between their persister numbers per well. If they do not correlate, diverse types of prime cells likely exist. To understand this, we did experiments similar to Fig. 4a, but we tested Replica 1 with Amp and Replica 2 with Apr. In this case, both replicas had a transient memory, and the memory was lost by 1:20 dilutions (Fig. 4d).

### Primed cells are not spontaneous persisters

Previous researchers proposed spontaneous persisters formation. This elegant hypothesis proposes that persisters can be generated stochastically at a constant rate during exponential phase growth and switch to a dormant or a protected state (distinct slower growth rate than other cells, and this slowed growth rate is maintained for several generations and turn into persisters in the presence of stress) (5, 21, 33). In addition, they proposed that during exponential growth, these phenotypic variants (e.g., persister formation) could also be induced by stress (5). However, several research groups criticized the concept of spontaneous persister formation (35, 39), questioning if it exists or is a proper terminology because spontaneous persisters were defined as dividing cells (5). In the original paper where persistence was proposed in 1944, persisters were defined as non-dividing cells (40). We currently use the original definition of persisters; they do not divide. In addition, if persister formation is only induced by stress, all cells in the population should turn into persisters instead of a small percentage of the population, or it should be random (no memory as we have shown here). In addition, induced persister formation (or sense-and-respond strategies) could be costly for the cells because it might necessitate constitutive expression of essential sensory machinery (41).

On the contrary, primed cells (already prepared for stress in a population through heterogeneity) could provide a simple mechanism for adaptation to stresses they might or might not encounter. A key phenotype of spontaneous persisters is that they grow slowly. However, we did not observe any significant growth rate changes among the clones, and recent results demonstrate that persisters are not slow growing before antibiotic treatment (42). To further explore whether prime cells are reliant on slow growth, we constructed a simple mathematical model (explained in the Methods), where we plotted the fraction of persister cells $\frac{y(t)}{x(t)}$ as a function of time for persister proliferation being 100%, 90%, 80%, 50%, and 0% of the proliferation rate of the drug-sensitive cells. We observed when persister cells do not proliferate ( $k_{p}=0)$ , their fraction rapidly dilutes back to the steady-state level in a few generations; Fig. 4e). These results show that a high correlation in persister maintenance, as seen in Fig. 4b for several generations, requires persister proliferation. For example, one requires $k_{p}=0.9k_{d}$ for it to take roughly four generations for the fraction of persisters to fall to 50% of its initial levels, similar to the drop in the correlation between replicates to 0.5 in Fig. 4b. Our evidence clearly shows that primed cells are not persisters (non-dividing cells) before antibiotic treatment, since they grow and maintain a transient memory. Although primed cells are not spontaneous persisters, it does not mean they are unrelated.

## DISCUSSION

Phenotypic heterogeneity is a fact of life and exists in both unicellular and multicellular organisms (1, 2, 4). This latent variation in phenotypic plasticity can be revealed in an unfavorable environmental condition (4). Noise in gene expression can drive this heterogeneity, and heterogeneity is hypothesized to be a key player that regulates bet-hedging strategies to endure harsh environmental fluctuation, such as bacterial persistence (2, 43). Persisters have reduced efficacy to antibiotic treatment and are a key contributor to the rise in antibiotic resistance. Unraveling the underlying molecular mechanisms of heterogeneity is, therefore, crucial to comprehend bacterial persistence.

In this study, we used a powerful FT framework to infer transient cell states that arise via reversible and non-genetic mechanisms, recently employed in probing cancer persistence (14, 17, 18), to find hidden features of bacterial persistence. Using the FTs, we tested the variation between clonal populations that originated from an identical clone and showed that a subset of the population, primed cells, have a “memory” that leads to high numbers of persisters. Our results demonstrate that under the same conditions, phenotypic heterogeneity often occurs in a range of ~60- to 100-fold and ~40- to 70-fold for Amp and Apr, respectively, despite (likely) being driven by a stochastic noise in gene expression. The relative consistency in primed cell numbers suggests that changes in specific genes result in primed cells, although we did not experimentally explore the mechanisms of primed cells in this work. Having high numbers of primed cells is a selective advantage that offers phenotypic plasticity to a bacterial population experiencing frequent harsh environmental stress. This heritable transient state might be favored in the course of evolution as a survival tactic compared to DNA mutation because it requires no long-term commitment. A recent paper demonstrated a transient cellular memory in *E. coli* and that inheritance of non-genetic elements can help maintain cellular memory (44), thus reducing variation among new cells for a few generations (44). They found that some inherited elements, cell size, and the time required for cell division were maintained for nearly 10 generations (44).

We provided a mathematical model that supports and provides clues to the regulation of transient memory, but we have not yet identified the regulatory mechanism(s) behind primed cells. We certainly do not want to propose any specific hypotheses for the regulatory mechanisms of prime cells because after over 80 y of research, the persister literature is scattered with assertions of “essential” genes for persisters. However, none thoroughly explains the persistence mechanism, and stating a mechanism for primed cells is premature. Moreover, it would only add more confusion to a field already jumbled with multiple proposed persistence mechanisms. The list is long, but here, we will showcase some examples of proposed mechanisms underpinning persistence and how primed cells may be related. The Balaban group has provided strong evidence that growth arrest is connected to persistence (33, 45), although, as we previously mentioned, primed cells are not spontaneous persisters. However, primed cells may be closer to growth arrest than susceptible cells. If this closeness to growth arrest were passed on for generations, it would match our observed results.

It was once thought and is still often cited that toxin-antitoxin (TA) systems are essential to all persister phenotypes. However, this is incorrect because a bacterial cell containing no known or hypothetical TA systems produces strong persister levels (38). Instead of being essential to persistence, TA systems can help regulate persisters. Primed cells may have a different ratio of toxin to antitoxin inside the cell, allowing them to enter persistence more readily than susceptible cells. If the TA ratio was passed down several generations, it could explain the epigenetic memory in primed cells. Previous work by the Balaban group hinted that TA systems could be important to persister variations (46). They probed the mode of action of HipBA, a TA system where HipA is the toxin and HipB is the antitoxin, by artificially overexpressing the HipA toxin. The more they increased the HipA toxin levels, the longer it took colonies on a plate to form (growth arrest increased). Interestingly, they observed that growth arrest occurred at two different time scales with 11-min and 220-min delays. They proposed that the antitoxin completely rescued some cells, and their growth was similar to that of cells where HipA was never artificially expressed. While in other cells, it took longer for HipB antitoxin to restore growth. Their results do not fit our findings because of the evidence we showed that primed cells are not spontaneous persisters and do not have an apparent growth arrest. In addition, because the majority of the cells (~99.9%) are not persisters (~0.1%), a significant time delay for them to grow (from 11 min to 220 min) would result in persister levels rapidly decreasing with each division. They would fall too quickly to see the transient memory we observe (Fig. 4e) (we assume, because it seems highly unlikely, that primed cells are not produced at an equivalent rate to those not dividing). However, as previously mentioned, this does not exclude primed cells from being close to growth arrest and passing this information on for generations. In order to determine if TA systems affect primed cells, a future experiment may be to look for primed cell formation in a cell lacking all TA systems, similar to what Hossain et al. (38) did.

The Wood group’s persuasive work has shown that ribosomal dimerization is important for persistence. The disruption of ribosomal dimerization in *E. coli*, e.g., an *rmf* knockout, results in reduced long-term survival rates during starvation and dramatically reduced persister levels but still results in persister cells (47). Because persister cells are still formed even if the cell lacks the capacity to dimerize ribosomes, this cannot be the essential mechanism of persisters. It is possible the expression of ribosomal dimerization genes are upregulated in primed cells (by some unknown means) and passed down several generations. This could explain the outcomes we observe. Primed cells could be related to TA systems, ribosomal dimerization, growth arrest, or something else. In each case, these options only provide an intermediate method (e.g., higher toxin level, higher dimerization, or closeness to growth arrest) to get to primed cells but not the mechanism that sparked their disruption.

We considered if primed cells were heteroresist cells, but this is not the case. Heteroresistance is an intriguing and poorly understood type of resistance where a phenotypically unstable and minority-resistant subpopulation (typically undetectable and caused by genetic mutation) co-exists with the susceptible population (48). For example, Choby et al. (48) showed that a small subpopulation contains beta-lactamases that, at low levels, are ineffective at resisting beta-lactams. However, through gene amplification (mutation) of these genes, cells can resist higher levels of beta-lactams. Interestingly, the heteroresistance pre-existed as a small population before antibiotics and can grow in the presence of antibiotics; however, they can return to the pre-selection frequency if grown over generations in the absence of the antibiotic (48). This phenotype is similar to what we observe with primed cells, but primed cells are not heteroresist cells caused by a change in DNA. Heteroresist cells are antibiotic resistant and will not be killed by the antibiotics over time, but primed cells continue to die with lethal antibiotic exposure. Primed cells do not grow in the presence of antibiotics, as we tested at different time points and persisters eventually decrease over time. In addition, high persisters formed from primed cells will be killed by the antibiotics over days of treatment, even in a highly nutrient-rich, buffered environment (data not shown), unlike heteroresist cells. Choby’s group diluted heteroresist cells (into 1:1,000 times/day, in total 1:10^18^ times) for 6 days to return to the pre-selection frequency, whereas primed cell memory is effectively lost with a 1:100 dilution. In addition, we show primed cells exist when treated with a combination of beta-lactam (Amp) and a ribosomal targeting antibiotic (Apr). An increase in the number of beta-lactamases that a cell produces will not allow cells to survive better against Apr. Our experiments show that primed cell levels correlate when splitting a culture and testing Replicate 1 with Amp and Replicate 2 with Apr (Fig. 4d). This experiment further supports that primed cells are not the heteroresist cells identified by the Choby’s group. It is defined as an unstable mutation, and the resistant subpopulations can revert to susceptibility in the absence of antibiotic stress within a limited number of generations. Although 50 generations is most often used as a limit (49) and the loss rates for heteroresistance are typically from 25 to 27 generations (49), we see a loss in about seven generations (1:100 dilution). This much quicker loss in survival levels means that we are looking at something different than the Choby’s group. We have proposed a change in the transcription level leads to primed cells. With the current evidence, we state that primed cells lead to persisters, not heteroresist cells, because no researchers have classified and provided experimental evidence that heteroresist cells can be caused by a non-genetic mechanism (caused by a non-mutation) (49), persister cells do not form through mutations, and primed cells that result in persisters are not resistant.

A few pathways for epigenetic memory related to stress survival are already known in bacteria. DNA methylation is a common method of epigenetic regulation in organisms, and many forms of methylation have been identified in bacteria (50 – 52). Some are related to antibiotic resistance or persistence (53, 54). For example, the deletion of *dam*, which is responsible for adenine methylation, can lead to lower persister numbers in pathogenic *E. coli* strains (55). Another recent study showed multisite phosphorylation may regulate the phenotypic variability in a bacterial population. A gene encoding ppGpp Synthetase, *sasA*, is regulated by multisite phosphorylation of WalR and exhibits elevated levels of extrinsic noise in gene expression. Due to this noise, cells having elevated levels of *sasA* expression have increased short-term antibiotic tolerance (13). Another possible mechanism could be ploidy. The Brynildsen group showed that ploidy or chromosome abundance produces phenotypic heterogeneity that affects persister numbers. Stationary-phase *E. coli* cells with two chromosomes had ~40-fold more persisters than cells with one chromosome against fluoroquinolone antibiotic (56).

We previously provided strong evidence that no single gene causes persistence, but disruption of networks leads to persistence (27). This assertion means that multiple genes could lead to persistence as long as the network disruption was substantial. Disruptions of essential cellular networks can lead to growth arrest, which aligns well with the Balaban group’s work on the connection between growth arrest and persistence (33, 45). This means there are likely several gene disruptions or causes of growth arrest that could lead to persistence. We hypothesize (like persisters) that multiple mechanisms could cause primed cells. Currently, we are working on the regulatory mechanism(s) of primed cells, and we do not propose any specific mechanism because this finding is entirely new, and multiple mechanisms could lead to primed cells.

In this study, we have not dealt with viable but nonculturable (VBNC) cells. Several studies showed VBNCs and persisters share similar phenotypes, and the major difference between them is that after stress (e.g., antibiotics), persisters can grow on Petri plates, while VBNCs can only grow in liquid. Currently, there is a furious debate if VBNCs are actually persisters, simply dying cells, or if VBNCs even exist (57 – 61). A recent study called into question many of the indicators that are currently used to classify bacterial cells as alive or dead (62).

We tested if VBNCs could be resurrected using the 0.5 cell/well technique. The 0.5 cell/well is based on cell counts on Petri dishes (CFU/mL). Often, VBNCs are cited as being at equal concentrations (using a hemocytometer and microscope) as CFUs (63, 64), but we did not observe this. We observed a ~20% count difference (cell/mL) between hemocytometer and agar plate count, which is within the hemocytometer’s manufacture error rate. However, still, if VBNCs are present and grow only in liquid, we expect 1 cell/well in a 96-well plate when using CFU/mL from Petri dishes. Instead, we consistently get ~48 wells with growth. We also consistently get growth in ~24, ~12, and ~6 wells when we use 0.25, 0.125, and 0.0625 cell/well, respectively, with an *r^2^* of 0.97, when plotting the wells with growth vs cells/well based on CFU/mL. Supplementing media with pyruvate may resurrect VBNCs quicker (65, 66). We tested the addition of pyruvate after antibiotic therapy with two carbon sources: glycerol and glucose. VBNCs did not resurrect; only ~48 wells had growth.

Recognizing that the previous assays do not account for all the nuances of VBNCs, we tested whether VBNCs would alter our overall results using 2 cells/well (based on CFU/mL). Again, we see a high variation with Amp treatment, ~78-fold (Fig. S1c). This shows that if there are VBNCs, they are not skewing our results and that we still observe primed cells even with 2 cells in each well. It also demonstrates that the FT does not require 1 cell/well, which is an important finding because it is unrealistic to assume that every well we test will always have 1 cell when diluted to 0.5 cells/well.

Overall, our findings show that a subset of the population has a transient memory allowing them to prepare for antibiotic stress; we refer to them as “primed cells.” We call these cells primed cells instead of persister cells for consequential reasons. The word “primed” means prepared or ready or conditioned for prompt action or use. These cells are prepared/ready and conditioned for prompt action to survive lethal antibiotic stress. Our results show that primed cells are not dormant (primed cells grow and divide, unlike persister cells), and they must divide because bacteria cannot have epigenetic memory passed down for generations unless cells are dividing. High primed levels lead to high persister levels over several generations. Primed cells are prepared and can hold a transient epigenetic memory, but the mechanism behind this memory is currently undefined. Further exploration is needed to determine the regulatory mechanism(s) behind this cellular memory and the contribution of non-genetic factors in phenotypic heterogeneity.

## MATERIALS AND METHODS

### Microbial strains and media

*Escherichia coli* DH5αZ1 having the p24KmNB82 plasmid was used in this study. We used this strain because DH5αZ1 was a derivative of *E. coli* K12 strain, and it has been used in our previous persistence studies (27, 28). The cultures were grown in the defined media MMB+ (27, 28) with thiamine (10 µg/mL 0.5% glycerol [or glucose]), Km (25 µg/mL), and amino acids (40 µg/mL) or on Miller’s lysogeny broth (LB) agar plates +Km (25 µg/mL). For pyruvate assays, MMB+ media were supplemented with 2 mM sodium pyruvate. All cultures were incubated at 37°C and shaken at 300 rpm.

### Population-level variation check (noise control)

We used two different types of antibiotics, Amp [target cell wall (31)] and Apr [target 30S ribosome (32)], for all FTs. We started with a mid-log phase *E. coli* culture having ~1E + 8 CFU/mL (~OD 0.5), subsequently divided the culture into 48-well (48 replicates) of 96-Well Optical-Bottom Plate with Polymer Base (ThermoFisher) and treated them with Amp (0.1 mg/mL) or Apr (0.1 mg/mL) for 3 h at 300 rpm, 37°C in a FLUOstar Omega microplate reader. Persister percentage was calculated by comparing CFUs per milliliter (CFU/mL) before antibiotic treatment to CFU/mL after antibiotic treatment. Plates were incubated at 37°C for 42–48 h and then scanned using a flatbed scanner. Custom scripts were used to identify and count bacterial colonies (67, 68).

### Fluctuation test from a single-cell level

We started with a log phase *E. coli* culture with ~1E + 8 CFU/mL (~OD 0.5) and subsequently diluted the culture into 0.5 cell/well in a 96-Well Optical-Bottom Plate and grown in a FLUOstar Omega microplate reader at 37°C, 300 rpm. Each dilution experiment was done twice for each single-cell experiment to confirm the dilution, and cultures were only picked when 50–60% of the well had growth (48–55 wells out of 96 wells). The single cell was proliferated to mid-log phase ~1E + 8 CFU/mL (~OD 0.5) and treated with Amp (0.1 mg/mL) or Apr (0.1 mg/mL) for 3 h, 300 rpm at 37°C. The persister percentage was calculated in the same manner described in the above section. The splitting and dilution test was done exactly as above, except when the single cell proliferated into mid-log phase, the cultures were separated and diluted into two plates with pre-warmed MMB+ media. Then, the cultures were grown to exponential phase, and persister assays were performed.

### Antibiotic resistance assay

After each FT, clones were diluted 1:100 in 1-mL LB media and were grown for 12 h. The cultures were then centrifuged for 3 min at 16,000 x *g*. After that, the supernatant was removed, and the pellet was streaked with and without antibiotic containing LB agar plate and incubated at 37°C.

### Minimal inhibitory concentration test

Clones, including high persister clones from FTs, were diluted 1:100 in 1-mL LB media and grown for 12 h. First, 0.2 mL of culture was spread on an LB agar plate, and then an MIC strip was placed on top of it and incubated at 37°C.

### Mathematical model

To understand prime cell proliferation rate, we considered a model of persister formation where drug-sensitive cells switch to a persister state with a rate $k_{1}$ , and persister cells revert back to the drug-sensitive state with a rate $k_{2}$ . We assumed the rate of cellular proliferation in the drug-sensitive and persisters states to be $k_{d}$ and $k_{p}$ , respectively. In an expanding cell colony, the number of persister cells can be captured by the following system of ordinary differential equations:


$$\frac{dx}{dt}=k_{d}x\left(t\right)-k_{d}y\left(t\right)+k_{p}y\left(t\right)$$

$$\frac{dy}{dt}=k_{p}y\left(t\right)+k_{1}x\left(t\right)-k_{1}y\left(t\right)-k_{2}y\left(t\right)$$

where x(t) and $y\left(t\right)$ are the total number of cells and the number of persister cells, respectively, at time t . By setting,


$$k_{1}=\frac{f_{s}\left(k_{2}+\left(k_{d}-k_{p}\right)\left(1-f_{s}\right)\right)}{1-f_{s}}$$

ensures that the steady-state persister fraction ${lim}_{t\to \infty }\frac{y\left(t\right)}{x\left(t\right)}=f_{s}$ . We considered a clone that initially had a high fraction of persister cells. We used the model to explore the relaxation of persister numbers back to steady-state levels, and how this time scale particularly depends on the persister proliferation rate $k_{p}$ . We then plotted the fraction of persister cells $\frac{y(t)}{x(t)}$ as a function of time for persister proliferation being 100%, 90%, 80%, 50%, and 0% of the proliferation rate of the drug-sensitive cells.

### Cell counting

Initially, using the common protocol for a hemocytometer, microscopic counting, and live/dead dye, we saw about equal “VBNCs” per non-VBNCs in log and stationary phase and with lethal antibiotics. This is consistent with the literature (63, 64). However, we adjusted our counting protocol to match the manufacturer’s recommendation. As a result, we no longer see two times the VBNCs compared to the CFU/mL; instead, the microscope count and Petri plate count only varied by ~20–25% in log phase.

We attempted to use several live-dead dyes to calculate viability after antibiotic treatment, but we found these assays to be quite unreliable, as recently cited in the literature (57, 69 – 72). So, we took the other approaches we described.

## SUPPLEMENTAL MATERIAL

Fig. S1 spectrum.01219-23-s0001.docx Supplement Figure 1 and legend.

ASM does not own the copyrights to Supplemental Material that may be linked to, or accessed through, an article. The authors have granted ASM a non-exclusive, world-wide license to publish the Supplemental Material files. Please contact the corresponding author directly for reuse.
