# Supplementary material for: Escherichia coli cells are primed for survival before lethal antibiotic stress
Source: Microbiol Spectr. 2023 Sep 12;11(5):e01219-23. doi: 10.1128/spectrum.01219-23 (PMC10581089; doi:10.1128/spectrum.01219-23)
Supplement: Fig. S1 — Supplement Figure 1 and legend. [file spectrum.01219-23-s0001.docx]

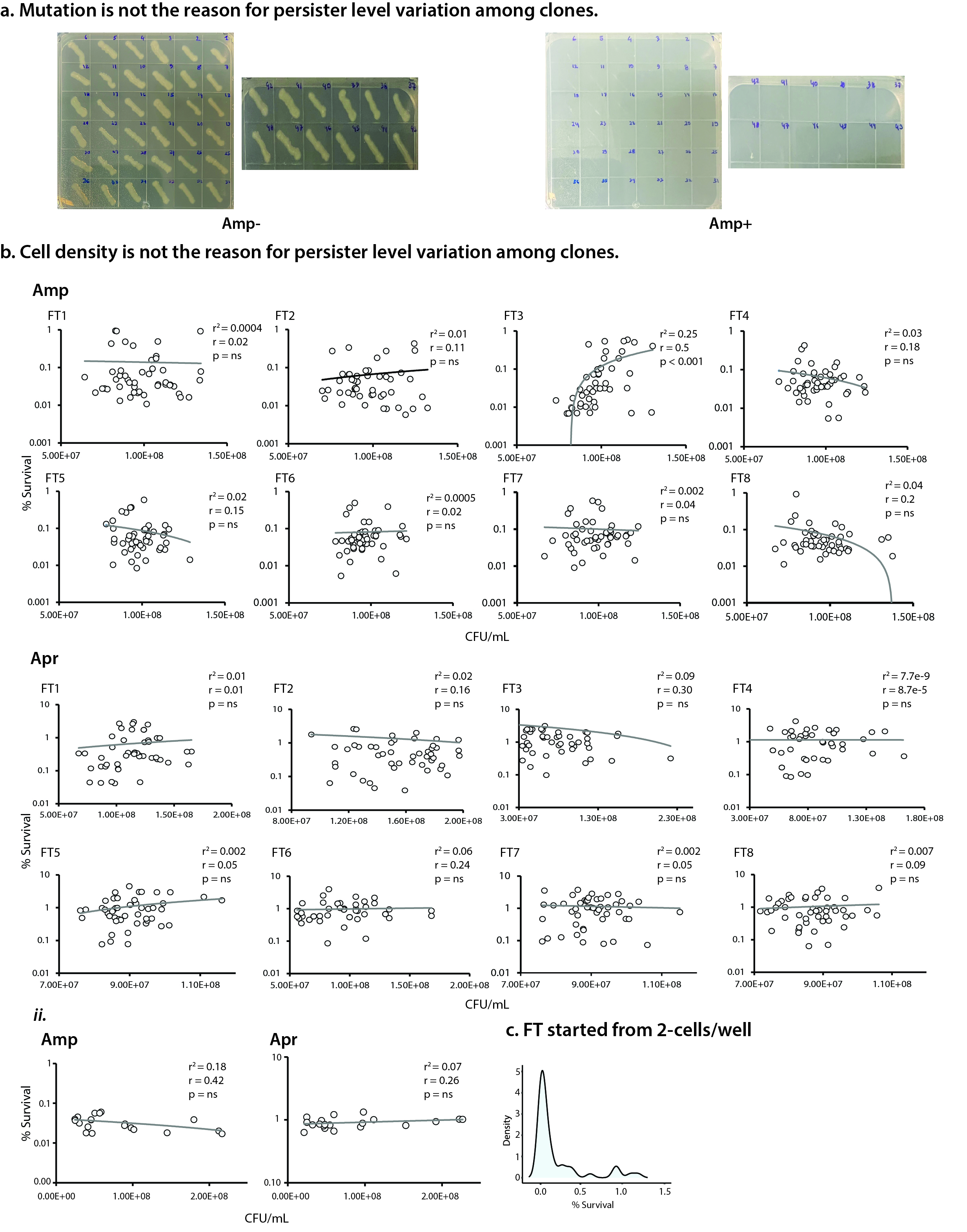


**Supplement Fig. S1. a.** Streaking plate shows mutation is not the reason for persister level variability among clones. **b.** Cell density is not the reason for persister level variation among clones. ***i.*** Each FT tests treated with Amp or Apr show no correlation between CFU/mL vs. % Survival. ***ii.*** General populations treated with Amp or Apr show no correlation between CFU/mL vs. % Survival. **c.** FT started with 2-cells/well, and showed ~78-fold variation among clones. Linear regression lines are shown in b.
